# Active Human Transposable Elements: Long-Read Sequencing Technologies, Computational Analysis, and Implications for Human Disease

**DOI:** 10.3390/biom16091247

**Published:** 2026-08-27

**Authors:** Dániel Vörösvácki, Nikolett Szakállas, Alexandra Kalmár, István Takács, Béla Molnár

**Affiliations:** 1 Department of Internal Medicine and Oncology, Semmelweis University, 1083 Budapest, Hungary; vorosvacki.daniel@phd.semmelweis.hu (D.V.);; 2 Department of Biological Physics, Eötvös Loránd University, 1053 Budapest, Hungary

**Keywords:** transposable elements, LINE-1, Alu, SVA, long-read sequencing, Oxford Nanopore Technologies, Pacific Biosciences, bioinformatics, epigenetics, structural variation

## Abstract

Transposable elements (TEs) account for nearly half of the human genome and shape chromatin organization, gene regulation, and genome evolution. However, their contributions to human physiology and disease remain incompletely understood. The most active elements in humans, LINE-1 (L1), Alu, and SVA, retain some copies with the ability to evade epigenetic repression and mobilize via target-primed reverse transcription (TPRT), whereas copies become inactive through various fragmentations and mutations. TE activity contributes to genomic instability and has been implicated in aging, cancer, neurological disorders, chromatin organization, and epigenetic regulation. Studying TE is challenging due to their repetitive and polymorphic nature. Recent advances in sequencing technologies and short- and long-read sequencing platforms, combined with specialized bioinformatic pipelines, currently enable more comprehensive characterization of TE insertions, deletions, expression, and epigenetic status. Computational approaches vary in sensitivity, specificity, and resource requirements, and their performance is influenced by sequencing modality, coverage, and the reference genome used. Assembly-based and read-based methods, as well as integrating methylation data or single-cell data, provide complementary insights into TE biology. This review summarizes the biology of active human TE, surveys state-of-the-art short- and long-read pipelines for TE analysis, and highlights their applications in studies of aging, cancer, and other complex diseases. We also provide practical guidance for selecting appropriate sequencing strategies and tools for TE-focused projects, and discuss emerging approaches and open questions in the field.

## 1. Key Messages

- Transposable elements are not merely “junk DNA”; some remain active and mobilize via target-primed reverse transcription, while others contribute to genome regulation and evolution.
- Long-read sequencing technologies, including Oxford Nanopore Technologies and Pacific Biosciences, provide improved resolution of repetitive genomic regions and enable characterization of complex TE insertions and structural rearrangements.
- Modern TE analysis pipelines integrate read-based, assembly-based, methylation-aware, and single-cell approaches to investigate TE insertions, expression, epigenetic regulation, and disease associations.
- Selection of an appropriate TE analysis workflow depends on sequencing modality, computational resources, the biological question, and the balance between sensitivity and specificity.

## 2. Introduction

Repetitive sequences account for a substantial fraction of eukaryotic genomes [1], with transposable elements (TEs) as the primary contributors [2,3]. In humans, approximately 45% of the genome is derived from TEs, and some estimates suggest that up to two-thirds of the genome may originate from highly fragmented ancestral TE insertions whose origins are no longer readily identifiable [4].

Recent advances in high-throughput sequencing technologies, particularly long-read sequencing platforms, together with increasingly sophisticated bioinformatic pipelines, have enabled more comprehensive characterization of the TE landscape [5]. These developments provide important insights into the evolutionary history, regulation, and functional impact of TEs in humans and other organisms.

Multiple TE classes have been identified in genomes analyzed to date and can be classified based on their propagation mechanisms and sequence homology [6]. A comprehensive overview of TE classification is beyond the scope of this review, which focuses primarily on active human TEs and their analysis using long-read sequencing approaches. Such information can be found in the reviews of human TEs [7]. For researchers new to the field, inconsistent TE nomenclature and overlapping classification systems may present a substantial challenge. Community resources such as TE Hub [8] provide useful summaries of current classification frameworks and TE-related reviews. Table 1 summarizes the major retrotransposon families that remain active in the human genome.

Recent telomere-to-telomere sequencing efforts and large-scale analyses of repetitive DNA suggest that most major human TE families have now been identified [16,17]. Accurate TE detection remains challenging, especially in short-read data, because the high copy number and sequence similarity of TEs complicate unambiguous mapping to their true genomic loci [18]. This review focuses on recent computational approaches and bioinformatic tools developed for TE analysis in human genomic datasets, with particular emphasis on long-read sequencing applications.

### 2.1. LINE-1

**Figure 1:**
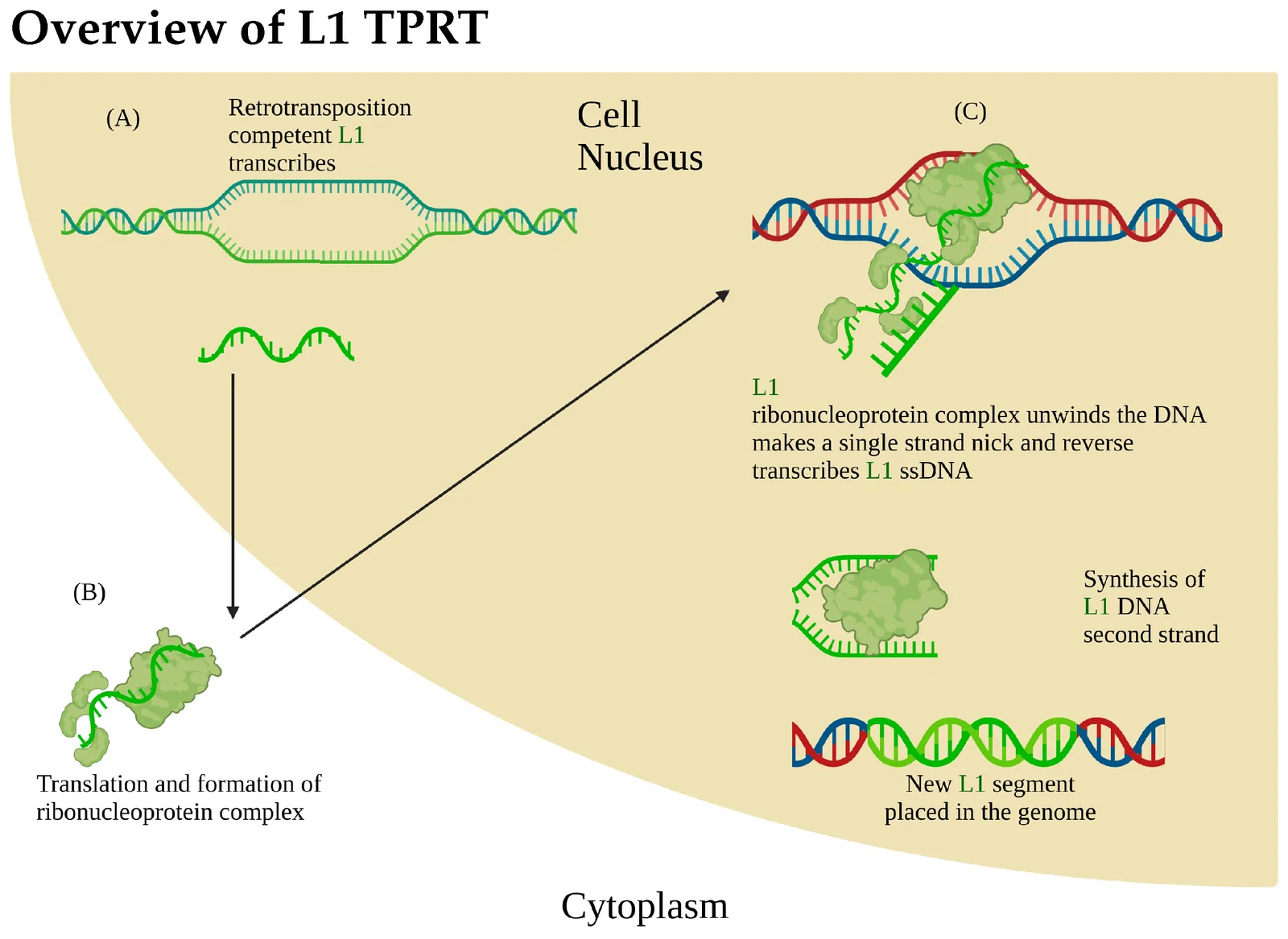
The schematic overview of L1-mediated TPRT. (**A**) L1 RNA is transcribed and exported from the nucleus. (**B**) L1 mRNA is translated to produce ORF1p and ORF2p, which form a ribonucleoprotein (RNP) complex, which is transported back to the nucleus. (**C**) The endonuclease activity of ORF2p nicks a genomic target site and the resulting 3′-OH group primes reverse transcription of L1 RNA leading to the integration of a new copy. Created in BioRender. Linkner, T. (2026) https://BioRender.com/g57plm7.

LINE-1 (L1) elements constitute approximately 17% of the human genome [3], and more than 30% of the genome reflects L1-driven activity through other retrotransposons that exploit the L1-encoded retrotransposition machinery [19,20,21]. L1 is the only currently active autonomous TE family in humans [19,20,21], with 500,000–900,000 L1 copies in various states of truncation and fragmentation according to different reference genomes and annotation methods. L1 mobilizes through target-primed reverse transcription (TPRT), as explained in Figure 1.

Nearly all active L1 copies characterized to date belong to the L1Pa1 lineage, also referred to as L1HS (human-specific) or L1TA (transcriptionally active). A small subset of these L1 elements, often termed “hot L1s” (3–9 copies per genome), accounts for approximately 80% of retrotransposition events [20]. Loss of L1 activity arises through multiple mechanisms, including 5′ truncation frequently caused by premature termination of TPRT, point mutations [18], nested insertions [22], and epigenetic repression such as hypermethylation. TPRT events frequently generate truncated L1 copies incapable of retrotransposition. On average, two human genomes differ by around 285 L1 insertions [23].

An intact L1Pa1 element is approximately 6 kb in length, comprises a 5′ untranslated region (UTR) containing an internal RNA polymerase II promoter, followed by three open reading frames (ORFs). ORF0 (213 bp) encodes a short peptide [24], ORF1 (1122 bp) encodes a 40-kDa RNA-binding protein, and ORF2 (3852 bp) encodes a 150-kDa protein with endonuclease and reverse transcriptase activity. ORF1p and ORF2p are essential for L1 retrotransposition, whereas ORF0p enhances retrotransposition frequency. Non-autonomous elements such as Alu and SVA rely on L1-encoded ORF2p for mobilization. L1 elements end with a 3′ UTR enriched in adenine and thymine bases.

Current estimates place the L1 germline retrotransposition rate at approximately one new insertion per 20–200 births, while short-read whole-genome sequencing in three-generation pedigrees suggests a rate of one insertion per roughly 63 births [25]. Insertions often occur at motifs that deviate from the canonical TTAAAA L1 endonuclease target site by up to two mismatches.

Because L1 is subject to strong epigenetic repression, most retrotransposition events occur in the germline when the genome is hypomethylated. New insertions are frequently generated by aberrant reverse transcription, as the transcription terminates before reaching the 5′ end of the L1 mRNA. Another phenomenon that reduces intact L1 is nesting, in which a new L1 copy integrates into a pre-existing element, damaging the pre-existing element. When L1 activity bypasses host repression, somatic retrotransposition occurs [18,26,27]. Somatic insertions typically feature a truncated 5′ end, an intact 3′ poly(A) tail, and flanking target site duplications (TSDs) of approximately 4–20 bp generated by L1 endonuclease during integration. Many bioinformatic pipelines identify somatic insertions by their absence in matched normal tissue DNA [28], although adjacent tissues may already exhibit early molecular alterations prior to overt tumorigenesis, potentially confounding detection [29].

### 2.2. Alu

Alu elements are non-autonomous, primate-specific retrotransposons [30] and account for approximately 11% of the human genome. With over one million copies present in various states of fragmentation, Alu elements represent the most numerous TE family by copy number. Intact Alu elements are approximately 280 bp long, are transcribed by RNA polymerase III, and rely on L1-encoded ORF2p for reverse transcription. Alu elements can undergo target-primed reverse transcription using ORF2p alone, whereas L1 elements require ORF1p. Alu likely derive from non-coding 7SL RNA, distinguishing it from most other SINEs, which generally originate from tRNA ancestors. Alu elements emerged approximately 65 million years ago [31], and current retrotransposition estimates range from one new insertion per approximately 20 births (phylogenetic inference) to one per approximately 40 births (short-read whole-genome sequencing) [3,12,25].

Alu retrotransposition has significantly shaped human evolution. At least 5% of alternatively spliced internal exons in the human genome originate from Alu sequences [32]. Like L1, Alu elements comprise hierarchical subfamilies in which older lineages (AluJ, AluS) have become mostly immobile due to truncation, mutation, or host repression, allowing younger subfamilies (AluY) to dominate current activity.

### 2.3. SVA

SVA (SINE–VNTR–Alu) elements are composite, non-autonomous retrotransposons [9] and constitute the youngest active TE family in humans. SVAs consist of a (CCCTCT)_n_ hexameric tandem repeat, a reversed Alu-like region, a GC-rich variable number tandem repeat (VNTR) domain, a SINE-R domain, and a poly(A) tail [9]. SVA elements range from roughly 700 bp to several kilobases in length, and some copies remain capable of retrotransposition. Their mobilization requires an intact L1 ORF2p and RNA polymerase II for transcription. The necessity of L1 ORF1p for TPRT remains contested and is likely subfamily- and assay-dependent. SVAs account for only approximately 0.15% of the human genome, with roughly 5100 annotated copies according to latest studies [9]. However, due to their variable-length structure, the number of full-length, potentially active SVAs remains difficult to determine accurately [26].

### 2.4. HERV-K

Human endogenous retrovirus K (HERV-K) represents the youngest endogenous retrovirus family in the human genome and retains several biologically active loci [33]. Although its current retrotransposition activity remains debated, and no confirmed disease-causing de novo HERV-K insertion has been reported to date [34], polymorphic insertions and transcriptional activity have been associated with several human diseases. Because this review focuses primarily on currently active non-LTR retrotransposons (L1, Alu, and SVA) and the computational methods developed for their detection, the biology of HERV-K is not discussed in detail. Some of the tools mentioned later natively handle HERV-K or can be configured to detect them.

## 3. TE Contributions to Human Health and Disease

One of the first disease-causing TEs identified in humans was an L1 insertion that caused insertional mutagenesis within exon 14 of the *F8* gene, resulting in hemophilia [35]. The earliest disease-causing Alu insertion was later detected in an intron of the *NF1* gene, where it disrupted normal gene function and caused neurofibromatosis [36]. TE insertions within exons or promoter regions often have deleterious effects on gene expression. As illustrated in Figure 1, TPRT involves endonuclease-mediated cleavage and can be associated with DNA damage and somatic insertions, providing a potential mechanism by which uncontrolled TE activity may contribute to genomic instability.

Some studies have shown that increased L1 ORF1p levels correlates with several types of cancer [37], ORF2p may also be associated with cancer because it is a protein with DNA nicking and TPRT activity, and any genomic instability could be carcinogenic. However, L1 ORF2p is difficult to measure, and only recently has it become possible to measure it reliably [38]. Some cancer tissues also exhibit a higher L1 insertion burden [18,39].

In vivo studies in mice have shown that L1-mediated activation of the cGAS–STING (cyclic GMP-AMP synthase–stimulator of interferon genes) pathway can accelerate aging in cardiovascular tissue [40]. Whether similar mechanisms contribute to human aging remains an open question.

Despite these detrimental effects, TEs also provide beneficial regulatory functions [41], shaping chromatin structure, gene expression, and genome evolution.

Higher-order chromatin organization is critical for long-range promoter–enhancer interactions and complex gene regulatory networks. CTCF (CCCTC-binding factor), a conserved architectural protein, binds directly to specific repetitive elements [42], and L1 elements have been implicated in topologically associating domain (TAD) organization and other aspects of three-dimensional genome architecture [43].

TE activity also contributes to somatic genome mosaicism in human brain tissue. For example, one study estimated an average of 13.7 L1 insertions per hippocampal neuron [44]. In contrast, another study estimated a somatic L1 insertion rate of 0.19 per cell and found no significant differences between hippocampal neurons and glia, cortical neurons, or AGS-derived hippocampal neurons [45]. While the exact number is contested, it has been hypothesized that this activity underlies aspects of neural plasticity required for synapse formation. In vivo experiments in mouse embryos have shown that L1 is involved in neural progenitor cell differentiation [46]. L1 elements may also take part in human brain development. A study has shown that increased L1 silencing results in reduced cerebral organoid development [47].

TE involvement is suggested in tissue regeneration and homeostasis: dental pulp stem cell-derived osteoblasts show lower TE methylation levels [48], and in axolotls, L1 reactivation occurs at the onset of limb regeneration [49], suggesting that loosening TE repression may accompany regenerative programs.

It is important to clarify that TE expression and hypomethylation correlations with cancer have been experimentally validated in human patients [6,37], while the beneficial effects listed above are supported by less robust evidence. The likely physiological functions shown here are still in the basic research phase and have potential applications. TEs appear to be part of complex regulatory networks. Such biological systems are likely easier to disrupt and damage than improve if they are carelessly interfered with.

### Regulation of TE Activity

As the previous segment explained, carefully controlled and regulated TEs can have many beneficial effects. This could be one of the reasons they occupy such a large percentage of the human genome. On the other hand, uncontrolled TEs pose a threat to genomic stability due to their potential for unchecked proliferation, illegitimate recombination, and the generation of DNA cuts during TPRT [18,37,50,51,52,53,54]. Consequently, host organisms have evolved multiple mechanisms to suppress TE activity. Additionally, immobile TEs can be domesticated, serving as scaffolds for chromatin organization and stability.

Genome-wide screens in the K562 cancer cell line identified numerous factors modulating L1 activity [55]. Hypermethylation of CpG islands within TE promoters is a major repression mechanism observed in differentiated tissues [50,56]. Tumor suppressors, such as P53, directly inhibit L1 transcription [54], while DNA repair factors (e.g., RAD51C, BRCA1/2, RAD54L) suppress TPRT [57]. The Human Silencing Hub (HUSH) complex, comprising TASOR, MPP8, and Periphilin, mediates L1 silencing in somatic cells via methylation-dependent chromatin condensation [58]. Genome-wide CRISPR knockout screens in K562 cells identified CBX1 and GA8PA as the strongest candidates for L1 activators and SRSF1 and RAD5 as the most likely candidates for L1 suppressors [55].

The Krüppel-associated box zinc finger protein (KRAB-ZFP) family provides another major host defense mechanism. Approximately two-thirds of KRAB-ZFPs recognize multiple sequences across diverse repetitive element families (LTRs, LINEs, SINEs, SVAs, simple repeats) [59]. Upon binding, KRAB-ZFPs recruit KAP1 (TRIM28) to induce sequence-specific transcriptional silencing through histone marking, maintaining genomic integrity. In germline cells, PIWI proteins and PIWI-interacting RNAs (piRNAs) guide cleavage of complementary TE transcripts, preventing transcription and mobilization [53].

Genes regulating nucleic acid metabolism, such as TREX1, SAMHD1, and ADAR1 have also been implicated in suppressing L1 mobilization [60]. TREX1 has been hypothesized to metabolize ssDNA products of TEs as part of the innate immune system [61]. SAMHD1 has several proposed models for restricting TE activity. SAMHD1 may restrict L1 retrotransposition by inducing depletion of L1 ORF2p. Nucleocytoplasmic shuttling is important for SAMHD1-mediated L1 suppression [62], and suggesting the nuclear transport needed for TPRT is also one possible point of L1 and TE regulation. SAMHD1 could also assist in the sequestration of the LINE-1 in cytoplasmic stress granules [63]. Furthermore some studies show ADAR1 can bind to L1 RNP and inhibit L1 RNP activity locally [64].

These intricate layers of TE regulation underscore the need for robust computational tools capable of accurately detecting, annotating, and interpreting TE insertions across diverse human tissues and diseases. Given these complex regulatory mechanisms, TEs may also hold promise as therapeutic targets or biomarkers, pending deeper mechanistic understanding.

## 4. Sequencing Techniques for TE Analysis

As discussed in the previous section, TEs play an important role in the genome and possess a complex regulatory system, and they could be used as prognostic or predictive biomarkers upon further understanding. This section reviews sequencing techniques, bioinformatic tools, and analytical pipelines developed to uncover TE insertions, deletions, genomic locations, disease associations, regulatory effects, and chromatin structure, and to support the planning and execution of future research.

Since the completion of the Human Genome Project [65], DNA sequencing has become significantly faster, cheaper, and more accessible. This rapid technological leap also allowed more specialized experimental methods to acquire more information not deducible from Sanger sequencing. The most widely used platforms in TE-focused sequencing studies are Illumina (IL), Oxford Nanopore Technologies (ONT), and Pacific Biosciences (PB). As many laboratories are bottlenecked by the type of sequencing platform available, this section presents the most used platforms.

Currently, the most widespread next-generation sequencing technology is based on sequencing by synthesis with fluorescently labelled nucleotides on the Illumina platform [66]. It has well-documented difficulties at properly mapping long repetitive sequences including TEs [67]. The difficulties arise from the fact that Illumina reads most commonly uses 2 × 150 bp paired-end reads. Thus, large repetitive sequences cannot be mapped unambiguously. This has led to a plethora of specialized techniques and tools. An independent comprehensive benchmark for TE detection in Illumina short-read data has been performed [68].

For choosing sequencing protocols, reagents and informatics pipelines combined with Illumina, SequenceEnG [69] could be a useful starting point. It is an interactive database of Illumina sequencing-based pipelines that is very helpful in selecting an analysis method for a specific task from a long list of possibilities, complete with methods and references. In the effort of making the bioinformatics of Illumina require less IT knowledge, many widely used tools are bundled together in the Galaxy online platform [70]. This makes TE research more accessible than approaches based on other sequencing platforms.

Third-generation sequencing (TGS) technologies have substantially improved TE analysis by enabling long-read sequencing, real-time signal acquisition, and detection of selected epigenetic modifications. The repetitive and fragmented nature of TEs creates substantial challenges for alignment and assembly algorithms [6]. Because many TE copies share near-identical sequences, short-reads frequently cannot be mapped unambiguously to a single genomic locus [71]. Long-read sequencing has been particularly transformative for the analysis of active human TE families such as L1, Alu, and SVA. These elements frequently generate polymorphic insertions, truncated copies, inversions, and nested retrotransposition events [22] that are difficult to reconstruct using short-read sequencing alone. Long-reads can span entire insertion loci together with their flanking genomic regions, enabling improved breakpoint resolution, more accurate genotyping, and characterization of insertion hallmarks including poly(A) tails, target-site duplications and complex structural rearrangements.

ONT platforms generate long-reads typically on the range of 10 kb or more [72], with ultra-long protocols producing reads exceeding hundreds of kilobases and, in some cases, reaching megabase scale. These long-reads enable spanning of entire TE insertions and other repetitive regions, reducing mapping ambiguity and improving structural variant (SV) detection. ONT sequencing operates by measuring changes in ionic current as native DNA or RNA molecules pass through nanopores, and the absence of PCR amplification also reduces amplification bias. This allows for preservation and direct detection of base modifications such as DNA methylation [73], making ONT particularly valuable for studying both TE insertions and their epigenetic regulation. Drawbacks include a higher raw error rate compared with short-read platforms [74], which can be mitigated by increased coverage, consensus polishing, or hybrid assembly strategies combining ONT and Illumina data.

The Pacific Biosciences (PacBio, PB) platform employs single-molecule real-time (SMRT) sequencing to generate long-reads, typically 10–25 kb, with highly accurate circular consensus reads (HiFis) reaching 99.9% accuracy [75]. PB is particularly advantageous for TE analysis because the combination of read length and accuracy allows confident detection of full-length TE insertions, SV, and complex rearrangements. SMRT sequencing can also indirectly detect epigenetic modifications, such as DNA methylation; combining SMRT with bisulfite sequencing [76] enabled studies on TE regulation. Limitations include higher per-sample cost and lower throughput compared with Illumina, which may be a bottleneck for large population studies.

Although ONT and PB both overcome many limitations of short-read sequencing for TE analysis, their strengths differ substantially. ONT provides substantially longer reads and direct detection of base modifications, making it particularly suitable for methylation-aware TE studies, resolving large repetitive regions, and detecting complex insertions spanning multiple kilobases [77]. In contrast, PacBio HiFi sequencing offers superior per-base accuracy, which improves breakpoint resolution and characterization of highly similar TE subfamilies. Consequently, the optimal platform depends on the primary biological question, sequencing budget, and required balance between read length, throughput, and nucleotide-level accuracy.

Despite their advantages, long-read technologies retain several limitations relevant to TE analysis. Ultra-long-sequencing protocols require high-molecular-weight DNA and careful sample preparation, which may not be feasible for archived or degraded samples [78]. In addition, long-read datasets typically require substantially greater storage capacity and computational resources during alignment, assembly, and polishing. For ONT data, sequencing chemistry and basecalling models may also influence methylation detection and insertion accuracy, complicating reproducibility between studies.

## 5. Bioinformatic Pipelines for TE Analysis

As described in the previous section, TEs play important roles in genome regulation, disease, and evolution. This section reviews sequencing techniques and computational pipelines developed to detect TE insertions and deletions, assess their genomic locations, quantify expression, and explore epigenetic regulation, providing guidance for experimental design and bioinformatic analysis.

**Figure 2:**
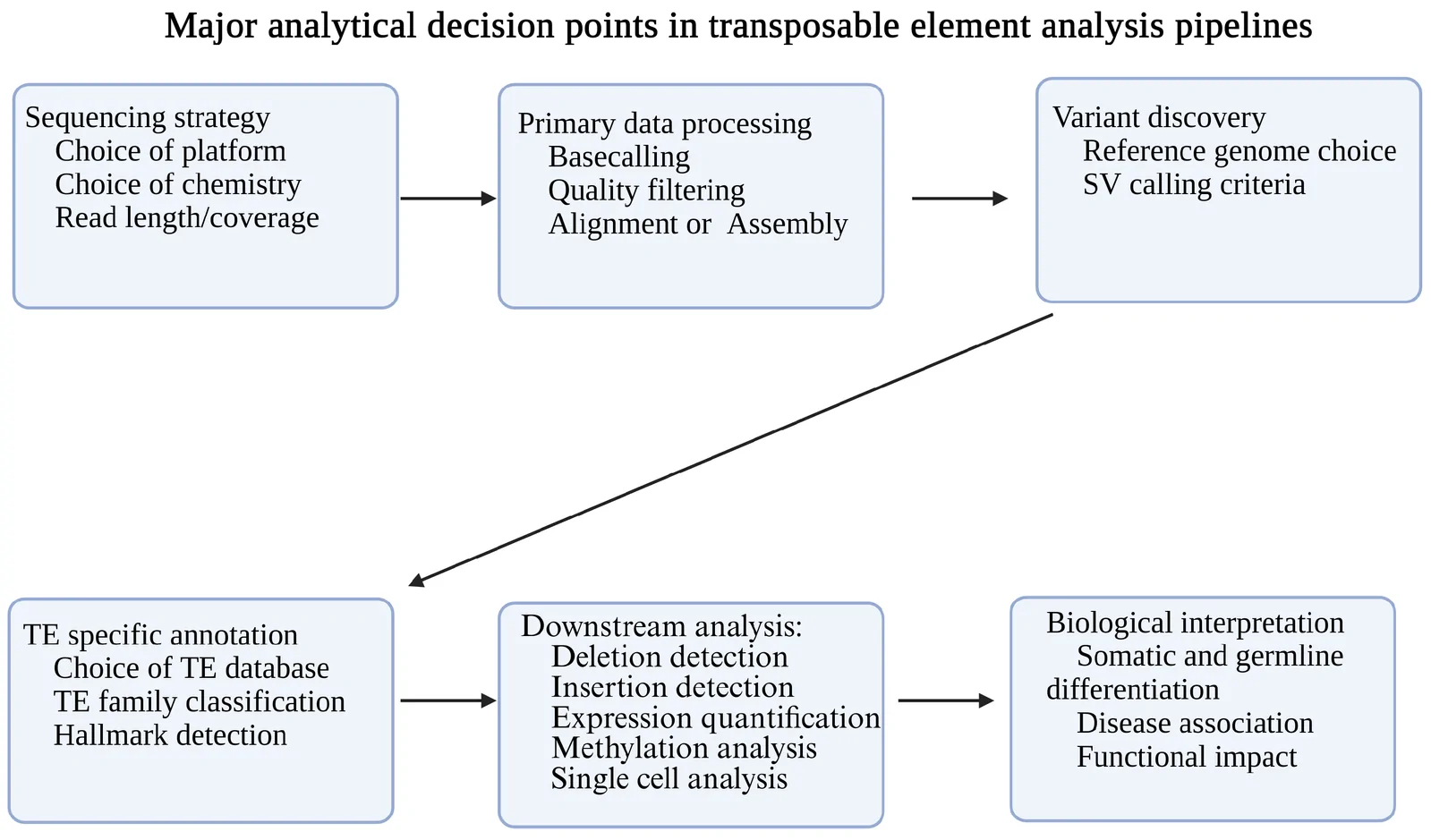
Conceptual framework for TE analysis using modern sequencing technologies, showing the major analytical decision points in TE analysis pipelines.

Fundamental questions in TE research include identifying the genomic locations of insertions, determining whether they disrupt genes or regulatory elements, and assessing their potential effects on gene expression. Addressing these questions is challenging because TEs are highly repetitive, many genomic copies are fragmented, and newly inserted elements are frequently structurally incomplete or aberrant [6]. While the main premise of these tools is highly similar, the differences lie in the types of questions the tools address and in where each tool is positioned within the analytical pipeline, as summarized in Table 2. It is also worth noting that while some of these tools do not directly answer these questions, it may be possible to use specialized R packages or other downstream analysis tools, measurements to answer questions such as: Are these insertions near specific genes? How many of these L1 insertion are full-length or have intact ORFs? How methylated are the insertions? Figure 2 shows the framework of TE analysis pipelines and the many possible changes, which makes comparison between studies challenging.

TE analysis must take the vast amount of fractured, no longer mobile, or otherwise inactive segments into account. The goal is to distinguish potentially active or polymorphic TE insertions and deletions from the large background of ancient, shared, and inactive TE-derived sequences present in the human genome. A common strategy in TE analysis is to identify sequence variants relative to a reference genome and subsequently annotate variants overlapping known TE sequences. By retaining only insertions absent from the reference genome and classified as TE-derived, the candidate search space can be substantially reduced. Although most TE detection pipelines follow this general principle, they differ considerably in how candidate insertions are identified, filtered, reconstructed, and validated.

### 5.1. Read-Based TE Insertion Detection

Read-based approaches operate directly on aligned sequencing reads and infer TE insertions from discordant alignments, clipped reads, split reads, or characteristic hallmarks of retrotransposition such as poly(A) tails and target site duplications. Because these methods avoid computationally intensive genome assembly, they are typically faster and require fewer computational resources. They are particularly suitable for studies involving large cohorts or heterogeneous tumor samples in which detecting low-frequency insertions may take priority over reconstructing full insertion architecture. However, increased sensitivity often comes at the expense of specificity, and read-based approaches may be more susceptible to false-positive predictions in highly repetitive genomic regions. Tools such as PALMER [79], xTea [80], sTELLeR [81], and TraDetIONS [82] primarily follow this strategy.

### 5.2. Assembly-Assisted TE Reconstruction

Assembly-based approaches attempt to locally or globally reconstruct the genomic sequence prior to TE annotation. By rebuilding insertion loci directly from sequencing reads, these methods can achieve improved breakpoint resolution and more accurate reconstruction of complex insertions, truncations, inversions, and nested retrotransposition events [22]. Such approaches are advantageous when validating potentially deleterious TE insertions or studying complex structural rearrangements. However, assembly quality is strongly dependent on sequencing depth and read length, and assembly-based workflows are generally computationally demanding due to the additional assembly and polishing steps. TELR [86] and TrEMOLO [87] are examples of assembly-assisted TE detection pipelines. Notably, TrEMOLO distinguishes between “insider” insertions incorporated into the genome assembly and “outsider” insertions supported only by aligned reads, highlighting the limitations of assembly completeness in repetitive regions and the differences between varied approaches.

### 5.3. Integrated End-to-End Workflows

A third category includes integrated, hybrid workflows that combine multiple analytical stages including alignment, SV calling, TE annotation, and visualization into unified pipelines. These integrated pipelines emphasize workflow standardization and simplified execution, although this may reduce flexibility for highly customized analyses. For example, Retroinspector [89] implements alignment, SV detection, annotation, and graphical summarization within a unified, script-driven workflow, whereas GraffiTE [88] combines SV discovery with TE-focused genotyping and annotation in a Nextflow [91] pipeline. Such integrated pipelines can reduce technical barriers for laboratories without specialized bioinformatics expertise but may provide less flexibility for custom-tailored analysis strategies.

Most current TE detection methods remain fundamentally library-based. Since the development of RepeatMasker [92], curated TE consensus databases, such as Repbase [17] and Dfam [93], have formed the basis of TE annotation pipelines. These approaches rely on sequence similarity and are highly effective for identifying previously characterized TE families. However, their performance depends heavily on the completeness and quality of the underlying TE libraries. This limitation becomes particularly important in non-human organisms where TE catalogs remain incomplete. Although machine learning approaches are increasingly being explored for TE classification and insertion detection, their adoption remains substantially more limited than library-based approaches. sTELLeR [81] is one example for complementing DBSCAN machine learning approaches with sequence similarity.

Despite the advantages of long-read sequencing, short-read sequencing data remain substantially more widely available in many research settings. Consequently, TE detection from short-read datasets remains highly relevant, particularly for retrospective analyses of existing clinical cohorts [71]. Large-scale benchmarking studies have demonstrated that specialized short-read pipelines can achieve robust TE detection performance in specific settings. For example, MELT [83] showed strong performance in exome sequencing datasets [68], whereas xTea demonstrated improved detection accuracy in whole-genome sequencing data [80]. These comparisons highlight that optimal pipeline selection is highly dependent on experimental design, sequencing modality, biological question being addressed, and the value of independent benchmarking.

Long-read sequencing has allowed the easier integration of TE analysis with epigenetic studies. ONT-based methylation-aware sequencing allows simultaneous characterization of TE insertions and their epigenetic state. Tools such as TLDR [90] integrate insertion detection with methylation profiling, enabling investigation of whether newly inserted elements remain epigenetically repressed or transcriptionally active. For those who have chosen a different pipeline and have methylation information available, using Modkit, Methylartist [94] or other methylation analysis in downstream analysis could complement insertion data with epigenetic information.

### 5.4. TE Expression Analysis Workflows

While the previous segment focused on the detection of TE insertional burden, deletions, and other SV, transcriptional profiling represents an equally important aspect of TE biology. Bulk RNA sequencing has been widely used to quantify TE expression, with tools including TEtranscripts [95], L1EM [96], Telescope [97] and SQuIRE [98]. A major computational challenge is the already mentioned extensive sequence similarity among TE copies. This is especially relevant to the youngest and most active segments, which causes many sequencing reads to map equally well to multiple genomic loci. Consequently, accurate locus-specific quantification remains substantially more difficult than family-level expression analysis, and benchmarking studies have demonstrated that multi-mapping can result in considerable locus-level false discovery rates. Comprehensive reviews and methodological comparisons of TE expression pipelines have recently been published and are therefore not discussed in detail here [99,100].

The use of long reads could substantially improve TE expression analysis by enhancing the reassignment of multimapping reads. LocusMasterTE is one such approach [101]. The authors reported improved mapping of the youngest and most active TEs compared with short-read RNA-seq tools. However, no independent benchmarking study of LocusMasterTE has yet to be completed. Because TE insertion detection has benefited from long-read technologies, expression-analysis pipelines may also achieve important methodological advances. At present, more advancements are needed for potential clinical applications.

Similarly, single-cell sequencing approaches are beginning to address cell-type-specific TE activity and somatic mosaicism. In heterogeneous tissues such as tumors or neuronal populations, bulk sequencing may obscure low-frequency TE insertions restricted to specific cell populations. CELLO-seq [102] extends long-read sequencing to single-cell TE expression analysis, while MATES [103] applies machine learning approaches to TE quantification in single-cell datasets. These methods remain computationally demanding but represent an important future direction for understanding TE dynamics at cellular resolution.

### 5.5. Computational Considerations

Computational requirements vary substantially between TE analysis strategies and are influenced by factors including alignment complexity, genome assembly, variant calling, methylation analysis, and machine learning integration. Read-based heuristic filtering approaches generally require fewer computational resources than assembly-based reconstruction methods [86,87]. Likewise, workflows incorporating methylation-aware basecalling, genome polishing, or single-cell analyses introduce additional computational overhead. Importantly, the computational complexity classifications provided in Table 2 represent qualitative methodological estimates rather than direct benchmarking measurements, as runtime and memory usage remain highly dependent on sequencing depth, read length, sample number, and available hardware infrastructure.

Most of the discussed tools are open source and typically installed from repositories through command line environment managers such as Conda, Mamba, or through the use of containers. Their use requires computational expertise, and analyses can be computationally demanding, particularly for long-read WGS data. A detailed summary of insertion detection tool availability, pipeline type, and organism scope is provided in Table 2, with download links listed in Data Availability Statement Section.

## 6. Practical Considerations and Guidelines for TE-Focused Studies

### 6.1. Literature and Tool Selection

This article is a narrative review and was not conducted as a systematic review. The relevant literature was identified through searches of PubMed and Google Scholar using combinations of terms including “Transposable Element detection,” “Mobile element detection,” “TE insertion detection,” “TE structural variation,” “TE methylation,” “TE expression analysis”. Tool selection was based on relevance to the detection and characterization of human TE insertions from sequencing data, with emphasis on methods published up to March 2026. Tools were included when they provided a distinct analytical strategy, supported long-read data, or represented an important comparator for TE detection in short-read data. As a narrative review, the presented tool collection is intended to illustrate the current methodological landscape rather than provide an exhaustive systematic inventory of all available software.

### 6.2. Selection of Appropriate TE Analysis Pipelines

This section presents a couple of possible scenarios and research questions and discusses what would be the optimal TE pipeline for that particular situation. Table 3 lists the different research objectives, their key considerations and some recommended tools for that task. These recommendations are intended as general guidance. Multiple tools may be suitable for a given study depending on sequencing technology, sample quality, computational resources, and desired balance between sensitivity and specificity. Table 2 highlights the necessary preprocessing steps needed for different tools, indicating the pipelines that use hard-wired TE consensus, which of them can be configured by the user, and most importantly, which sequencing platforms they support. While not an exhaustive list, this table should help new research groups entering the field.

#### 6.2.1. Retrospective Analysis of Different Cohorts

Research question: Which non-reference TE insertions are associated with a disease across hundreds or thousands of genomes?

Methodological considerations:

Large cohorts such as The Cancer Genome Atlas or the Pan-Cancer Analysis of Whole Genomes [14,104] often contain sequencing data generated using different platforms and library preparations. Therefore, compatibility across sequencing technologies, scalability, and standardized output become more important than reconstructing every insertion in maximal detail.

Recommended analytical strategy:

When both long-read and short-read samples are available, tools supporting multiple sequencing technologies, such as xTea, are well suited for these analyses because they can process both Illumina and long-read datasets using a unified analytical framework. When only short-read data were available, MELT demonstrated strong performance in independent benchmarking of exome sequencing datasets [68]. There have been successful breakthroughs with large-scale genomics studies involving TE; Rodriguez-Martin et al. [84] have performed one of the largest-scale TE detection studies to date in the framework of the Pan-Cancer Analysis of Whole Genomes project using TraFiC-mem. Their methodology could also serve as guidance.

#### 6.2.2. Clinical Validation

Research question: Does a patient carry a pathogenic TE insertion affecting a disease-associated gene?

Methodological considerations: In clinical investigations, confidence in individual insertion calls is generally more important than processing speed. Assembly-assisted reconstruction can improve breakpoint resolution and facilitate downstream validation. Extra attention must be paid for experimental verification as well.

Recommended analytical strategy: The most important caveat here is that no established gold standard or formally validated diagnostic pipeline exists as of the writing of this article for TE detection pipelines. If such study were undertaken, a haplotype-resolved assembly on an enriched long-read sample, annotated with a human-specific annotation tool and combined with experimental validation such as PCR, would be a good place to start.

#### 6.2.3. Tumor-Normal Comparison

Research question: Which TE insertions are somatically acquired during tumor development?

Methodological considerations: The analytical workflow should distinguish germline from somatic insertions while accounting for tumor heterogeneity.

Recommended analytical strategy: TraFiC-mem and TraDetIONS were specifically developed for paired tumor-normal analyses, and therefore, directly address this experimental design.

#### 6.2.4. Epigenetic Regulation

Research question: Are the TE insertions epigenetically silenced?

Methodological considerations: DNA methylation information must be available in addition to the sequence.

Recommended analytical strategy: TLDR was designed for this research question. Furthermore, combining other tools with downstream methylation analysis using Modkit or Methylartist [94] could also address the research question.

#### 6.2.5. Single-Cell Biology

Research question: Which cell populations express TEs?

Difficulty: Bulk sequencing averages signals across cells. Cell-level expression data is lost.

Recommended analytical strategy: Single-cell approaches that preserve cell identity are recommended, particularly single-cell barcoding combined with expression analysis. CELLO-seq or MATES are appropriate pipelines developed to solve this question.

#### 6.2.6. Structural Characterization

Research question: Are the TE insertions truncated, inverted, or associated with 3′ transduction events? Do they have intact open reading frames? Are they potentially active or aberrant?

Methodological consideration: Beyond localization, structural characterization is required.

Recommended analytical strategy: MEIGA-PAV was designed to address this research question.

#### 6.2.7. Low Tumor-Purity Analysis

Research question: Where are the TE insertions located in a liquid biopsy sample or other low tumor-purity sample?

Methodological considerations: The true signal, like cancer-relevant insertions, need to be filtered out from a potentially very small portion of the reads. Careful compromises must be made between precision and sensitivity.

Recommended analytical strategy: Similarly to clinical validation, no currently adopted standard practice exists. Every option to reduce the error rate and to increase the signal-to-noise ratio should be considered. Options such as ONT adaptive sequencing, to enrich the target sequences [105], or using Unique Molecular Identifiers (UMIs) [106], to reduce error rate, have the potential to make such studies more effective. Combining those with an adjustable-read-based tool could detect an insertion that would not appear in a tool not sensitive enough, like one based on de novo assembly.

No single workflow is optimal for all research objectives. Pipeline selection should primarily be driven by the biological question, sequencing modality, desired sensitivity, available computational resources, and downstream analyses rather than by overall tool popularity.

Finally, it is important to remember that the accuracy of TE detection depends strongly on sequencing quality. Read length, sequencing depth, N50, alignment quality, and basecalling accuracy influence both sensitivity and precision. Consequently, appropriate quality control should precede any biological interpretation.

### 6.3. Current Limitations of TE Analysis

Despite major advances in sequencing technologies and computational methods, several important limitations continue to constrain TE analysis. These limitations affect detection accuracy, reproducibility, computational requirements, and biological interpretation.

In human genomes, curated databases such as Dfam 3.9 and Repbase are generally assumed to cover the vast majority of currently recognized human TE families in the era of telomere-to-telomere sequencing. However, Repbase is now under commercial license, which may remain a practical barrier for some laboratories and institutions, especially in resource-limited regions. In non-human organisms, incomplete TE annotation and limited availability of curated TE libraries remain substantial challenges and may reduce detection sensitivity [107,108]. Consequently, researchers must carefully select the TE consensus libraries used during analysis, particularly when using tools restricted to predefined TE families. TE insertions absent from the selected database cannot be detected. Conversely, tools hardwired to broad databases, such as Retroinspector with the Dfam 3.9 human library, may recover large numbers of inactive or highly fragmented TE copies that are not biologically relevant for a given study, increasing downstream filtering requirements and computational burden.

Another important limitation is that many TE analysis pipelines were developed for highly specific research questions. Default parameters are therefore not universally applicable, as hyperparameters such as minimum read support, alignment thresholds, or SV filtering criteria can substantially influence sensitivity and precision depending on sequencing depth, tumor purity, and experimental design [80,81]. For example, highly heterogeneous tumor samples or samples with low tumor purity may require relaxed filtering thresholds to recover low-frequency somatic insertions, whereas clinical diagnostic workflows may prioritize specificity and reproducibility over maximal sensitivity. Sensitivity estimates reported by different tools are often difficult to compare directly because benchmarking datasets, sequencing depth, read lengths, and validation criteria vary substantially between studies.

A major unresolved issue in the field is the lack of comprehensive independent benchmarking studies across recently developed tools. Existing evaluations are often performed by the developers themselves and commonly compare new methods only against older approaches rather than against the most recent alternatives [80,81,88]. Although early independent benchmarking efforts for long-read TE detection tools such as PALMER, TLDR, sTELLeR, and xTea have begun to emerge, the rapidly expanding number of pipelines highlights the need for larger standardized benchmarking studies. Such efforts would improve reproducibility, clarify optimal use cases for each tool, and facilitate more objective selection of analysis workflows.

Computational reproducibility also remains challenging. Although most TE analysis tools are publicly available and open source, practical implementation often requires substantial bioinformatic expertise. Installation may be complicated by dependency conflicts, outdated packages, variable documentation quality, or incompatibilities between software versions. In addition, many pipelines rely on whole-genome sequencing data and therefore require substantial storage capacity, memory allocation, and wall-clock runtime. These technical barriers may limit adoption in smaller laboratories lacking dedicated computational infrastructure.

Reference genome selection introduces an additional source of bias. As illustrated in Table 4, substantial differences exist between commonly used human reference genomes such as GRCh38 and T2T assemblies. TE insertions identified relative to one reference genome may represent common polymorphisms absent from another assembly rather than novel insertion events. This issue is particularly relevant in underrepresented populations, where population-specific germline variants may be incorrectly interpreted as rare or disease-associated insertions if they are not represented in the reference genome. Potential solutions to this problem include the Human Pangenome reference [109]. Using their data could help in deciding if a detected non-reference TE insertion is a true somatic insertion or a subpopulation-specific TE.

## 7. Future Directions and Open Questions

Understanding the contributions of currently active and replicating TEs requires careful investigation of their role in disease. Transcript levels of Alu and L1 ORF1p [30,51] correlate with disease severity and may serve as biomarkers of poor prognosis. These observations suggest that TE activation may contribute to disease progression, although causality remains incompletely established.

The broader adoption of long-read sequencing, along with improved bioinformatic pipelines and experimental approaches specifically designed for WGS and TE detection, is expected to improve our understanding of the mechanistic links between TEs and human diseases in the near future. Integrating long-read sequencing, functional studies, and standardized analysis pipelines will be essential to fully understand TE biology and their roles in human health and disease.

At the time of writing, many of the listed TE analysis tools reviewed here have not been independently benchmarked. Such benchmarking could establish community standards for TE insertion detection, facilitate reproducibility, and provide developers with critical feedback for improving and harmonizing analysis pipelines.

Pangenome references and graph-based genome representations are already available [109,110,111], but their application in TE analysis remains underutilized. Other advancements that could support TE analysis include graph genome representations, standardized benchmark datasets, improved TE annotations, increasingly integrated long-read sequencing workflows, direct native RNA sequencing [112], advances in machine learning, and single-cell multiomics approaches. While several of these technologies are already established, their broader adoption and integration into TE research represent important opportunities for future methodological development. Together, these advances could help shift TE research beyond the identification of insertion sites toward addressing broader biological and clinical questions.

## 8. Conclusions

TEs have profoundly shaped the human genome, participating in an ongoing evolutionary dynamic as hosts evolve mechanisms to suppress their activity. Although most TE subfamilies have lost their ability to mobilize and now persist as genomic fossils, their continued presence underscores their biological significance. Future studies may clarify how TE activity contributes to aging, elucidate the mechanisms underlying the association between L1 expression and cancer, and determine whether TE activity can be therapeutically targeted.

## Figures and Tables

**Table 1:** Short summary of active mobile elements in the human genome. Length values are approximate and may vary between different subfamilies. Estimates for some values differ substantially between methods. For the Non-AUG translation of SVA, the evidence is limited. * The retrotransposon-competent SVA number is still not conclusively determined; the value of 1927, representing the number of full-length SVA elements detected in GRCh38 by Chong et al. [9], should be treated as an upper bound without accounting for mutations, epigenetic suppression and other possible mechanisms preventing SVA TPRT.

Name	L1	Alu	SVA [9]
Length (bp)	6000	280	700–4000
Copies in genome	500,000 [10] −919,967 [11]	1,100,000 [12]	3000 [13]–5100 [9]
Retrotransposition competent elements	80 [10]–114 [14]	≥852 [12]	1927 *
Autonomy	Autonomous	Dependent on L1	Dependent on L1
Transcribed	Yes	Yes	Yes
Translated	ORF0, ORF1, ORF2	No	Non-AUG translation [15]
Required for transposition	ORF1p, ORF2p	ORF2p	ORF1p, ORF2p

**Table 2:** Conceptual classification of long-read TE detection tools according to workflow architecture, preprocessing requirements, computational complexity, and organism specificity.

Tool	ONT	PB	IL	Strategy	Scope	Preprocessing	Complexity	Organism Coverage	Key Strategies/Primary Application
Alignment and read-based approaches									
PALMER [79]	✓	✓	×	Read-based	Detection only	Alignment	Medium	Human-focused	Detection of human TE families (L1, Alu, SVA, HERV-K) using long-read sequencing. Detects hallmark features of retrotransposition.
xTea [80]	✓	✓	✓	Read-based	Detection + genotyping	Alignment	Medium	Adaptable	Multi-platform TE insertion detection supporting short-read and long-read sequencing with machine learning-based genotyping.
sTELLeR [81]	✓	✓	×	Read-based	VCF annotation	Alignment + SV calling	Low	Adaptable	Lightweight TE insertion detection utilizing DBSCAN with low computational requirements and fast runtimes.
TraDetIONS [82]	✓	×	×	Read-based	Somatic TE insertion detection	Alignment + SV calling	Medium	Human-focused	Detection of somatic and germline TE insertions in ONT tumor-normal paired samples with TPRT hallmark detection.
MELT [83]	×	×	✓	Read-based	Detection	Alignment	Low	Human-focused	Benchmark detection tool for short-read sequencing of L1, Alu and SVA.
TraFiC-mem [84]	×	×	✓	Read-based	Somatic TE insertion detection	Alignment	Low–Medium	Human-focused	Pan-cancer somatic TE insertion detection tool for Illumina data.
MEIGA-PAV [85]	✓	✓	×	Hybrid SV analysis	Complex rearrangement analysis	Alignment + Variant calling	High	Human-focused	Detection of complex TE-associated rearrangements including inversions and potentially active L1 elements with intact ORFs.
Assembly-based approaches									
TELR [86]	✓	✓	×	Assembly-based	Detection + local assembly	Alignment	High	Adaptable	High-precision reconstruction and annotation of non-reference TE insertions using local assembly and polishing.
TrEMOLO [87]	✓	✓	×	Assembly-based	Integrated TE analysis	Assembly + alignment	High	Adaptable	Assembly-supported TE characterization. Distinguishes “insider” and “outsider” TE insertions using assembled genomes and aligned reads with graphical summaries.
Hybrid and end-to-end workflows									
GraffiTE [88]	✓	✓	×	Hybrid	Full pipeline	Alignment + optional assembly	Medium–High	Adaptable	Flexible TE-associated SV detection and genotyping pipeline supporting batch analysis and multiple input types.
Retroinspector [89]	✓	×	×	Read-based	Full workflow + visualization	Raw reads or alignment	High	Human-focused	Integrated workflow combining alignment, SV calling, TE annotation and built-in visualization/report generation.
TLDR [90]	✓	×	×	Read-based	Methylation-aware detection	Alignment	Medium–High	Adaptable	Simultaneous TE insertion and methylation analysis using ONT long-read sequencing.

**Table 3:** Practical guidance table on which TE analysis tools are useful for different research objectives. It is very likely that several other questions can be answered by these tools. At present, clinical validation has no gold standard and any such study should complement it with experimental validation.

Research Objective	Key Requirement	Suitable Approaches
Population cohorts	Scalability, multi-platform support	xTea [80], TraFiC-mem [84]
Clinical validation	High confidence	Human-focused tool with PCR or other experimental validation
Tumor-normal-pairing	Somatic comparison	TraDetIONS [82], TraFiC-mem [84]
Using existing Illumina data	Short-read support	MELT [83], xTea [80]
Methylation analysis	Native ONT reads	TLDR [90], other ONT compatible tool combined with Methylartist [94] or Modkit
Bulk expression analysis	RNA expression data	TEtranscripts [95], SQuIRE [98]
Single-cell expression analysis	Single-cell level expression data	CELLO-seq [102], MATES [103]
Structural characterization	Complex insertions	MEIGA-PAV [85]
Exploratory analysis	High sensitivity, built-in downstream analysis	Retroinspector [89], GraffiTE [88]

**Table 4:** TE content of the GRCh38 and T2T reference genomes. Data from [16] are rounded. Almost half of the genome is measurably taken up by TEs. Some estimates hypothesize that up to two-thirds of the genome consisted of TEs, but became so fragmented that they no longer readily recognizable as TEs [4]. The T2T reference genome contains around 200 Mb more base pairs in difficult to map regions. These differences highlight the importance of reference selection in experiments as some regions detected in T2T might not show up if the sample was aligned against the GRCh38 reference. Care must be taken when evaluating or comparing results using different reference genomes.

TE	GRCh38%	GRCh38 Mbp	T2T%	T2T Mbp
L1	17.36	507	16.77	512
Other LINE	4.07	118	3.90	119
Alu	10.43	304	10.09	308
Other SINE	2.80	81	2.68	82
SVA	0.15	4.50	0.15	4.65
LTR	9.16	267	8.84	270
DNA transposon	3.71	108	3.58	109

## Data Availability

No new data were created or analyzed in this study. Data sharing is not applicable to this article. The source codes for most bioinformatic tools discussed in this review are publicly available at the following repositories: CELLO-seq: https://github.com/MarioniLab/CELLOseq (accessed on 24 August 2026); GraffiTE: https://github.com/cgroza/GraffiTE (accessed on 24 August 2026); L1EM: https://github.com/FenyoLab/L1EM (accessed on 24 August 2026); LocusMasterTE: https://github.com/jasonwong-lab/LocusMasterTE (accessed on 24 August 2026); MATES: https://github.com/mcgilldinglab/MATES (accessed on 24 August 2026); MEIGA-PAV: https://github.com/MEIGA-tk/MEIGA-PAV (accessed on 24 August 2026); MELT: https://melt.igs.umaryland.edu/downloads.php (accessed on 24 August 2026); PALMER: https://github.com/WeichenZhou/PALMER (accessed on 24 August 2026); Retroinspector: https://github.com/javiercguard/retroinspector (accessed on 24 August 2026); sTELLeR: https://github.com/kristinebilgrav/sTELLeR (accessed on 24 August 2026); SQuIRE: https://github.com/wyang17/SQuIRE (accessed on 24 August 2026); TEtranscripts: https://github.com/mhammell-laboratory/TEtranscripts (accessed on 24 August 2026); Telescope: https://github.com/mlbendall/telescope (accessed on 24 August 2026); TELR: https://github.com/bergmanlab/TELR (accessed on 24 August 2026); tldr: https://github.com/adamewing/tldr (accessed on 24 August 2026); TraDetIONS: https://github.com/panummi/TraDetIONS (accessed on 24 August 2026); TraFiC-mem: https://gitlab.com/mobilegenomesgroup/TraFiC (accessed on 24 August 2026); TrEMOLO: https://github.com/DrosophilaGenomeEvolution/TrEMOLO (accessed on 24 August 2026); xTea: https://github.com/parklab/xTea (accessed on 24 August 2026).

## Abbreviations

The following abbreviations are used in this manuscript:

HERV-K	Human endogenous retrovirus K
HiFi	Highly accurate circular consensus reads
IL	Illumina
L1	Long interspersed nuclear element-1
ONT	Oxford Nanopore Technologies
ORF	Open reading frame
PB	Pacific Biosciences
RNP	Ribonucleoprotein
SMRT	Single-molecule real-time
SV	Structural variants
SVA	SINE-VNTR-Alu
TAD	Topologically associating domain
TGS	Third-generation sequencing
TE	Transposable element
TPRT	Target-primed reverse transcription
TSD	Target site duplication
UMI	Unique molecular identifiers
UTR	Untranslated region
VNTR	Variable number tandem repeat
WGS	Whole-genome sequencing
